# Systems Metabolic Alteration in a Semi-Dwarf Rice Mutant Induced by *OsCYP96B4* Gene Mutation

**DOI:** 10.3390/ijms21061924

**Published:** 2020-03-11

**Authors:** Limiao Jiang, Rengasamy Ramamoorthy, Srinivasan Ramachandran, Prakash P. Kumar

**Affiliations:** 1 Department of Epidemiology and Biostatistics, MOE Key Lab of Environment and Health, School of Public Health, Tongji Medical College, Huazhong University of Science and Technology, 13 Hangkong Road, Wuhan 430030, China; 2 Department of Diagnostic Radiology, Yong Loo Lin School of Medicine, National University of Singapore, 5 Lower Kent Ridge Road, Singapore 119074, Singapore; 3 Department of Biological Sciences, Faculty of Science, National University of Singapore, Singapore 117543, Singapore; ram10375@u.nus.edu; 4 Temasek Life Sciences Laboratory, 1 Research Link National University of Singapore, Singapore 117604, Singapore; sri@tll.org.sg; 5 NUS Environmental Research Institute (NERI), 5A Engineering Drive 1, Singapore 117411, Singapore

**Keywords:** rice, dwarfism, *OsCYP96B4*, metabolomics, NMR, qRT-PCR

## Abstract

Dwarfism and semi-dwarfism are among the most valuable agronomic traits in crop breeding, which were adopted by the “Green Revolution”. Previously, we reported a novel semi-dwarf rice mutant (*oscyp96b4*) derived from the insertion of a single copy of *Dissociator (Ds)* transposon into the gene *OsCYP96B4*. However, the systems metabolic effect of the mutation is not well understood, which is important for understanding the gene function and developing new semi-dwarf mutants. Here, the metabolic phenotypes in the semi-dwarf mutant (M) and ectopic expression (ECE) rice line were compared to the wild-type (WT) rice, by using nuclear magnetic resonance (NMR) metabolomics and quantitative real-time polymerase chain reaction (qRT-PCR). Compared with WT, ECE of the *OsCYP96B4* gene resulted in significant increase of γ-aminobutyrate (GABA), glutamine, and alanine, but significant decrease of glutamate, aromatic and branched-chain amino acids, and some other amino acids. The ECE caused significant increase of monosaccharides (glucose, fructose), but significant decrease of disaccharide (sucrose); induced significant changes of metabolites involved in choline metabolism (phosphocholine, ethanolamine) and nucleotide metabolism (adenosine, adenosine monophosphate, uridine). These metabolic profile alterations were accompanied with changes in the gene expression levels of some related enzymes, involved in GABA shunt, glutamate and glutamine metabolism, choline metabolism, sucrose metabolism, glycolysis/gluconeogenesis pathway, tricarboxylic acid (TCA) cycle, nucleotide metabolism, and shikimate-mediated secondary metabolism. The semi-dwarf mutant showed corresponding but less pronounced changes, especially in the gene expression levels. It indicates that *OsCYP96B4* gene mutation in rice causes significant alteration in amino acid metabolism, carbohydrate metabolism, nucleotide metabolism, and shikimate-mediated secondary metabolism. The present study will provide essential information for the *OsCYP96B4* gene function analysis and may serve as valuable reference data for the development of new semi-dwarf mutants.

## 1. Introduction

Rice (*Oryza sativa* L.) is an important staple food for more than half of the global population [1]. Its sustainable production is critical to the world’s food security and the health of the ever-increasing global population. Although nitrogen fertilization is essential for improving grain yield, the increasing usage rate will lead to increased plant height [2,3]. The resulting taller plants are prone to lodging caused by wind/rain, which will increase the difficulty of harvest, promote pre-harvest germination and fungal contamination, and ultimately lead to significant reduction in grain yield and quality [2,4].

As adopted by the “Green Revolution” [2], dwarfism and semi-dwarfism are among the most valuable agronomic traits in crop breeding [5,6]. The semi-dwarfism in rice enhances their lodging resistance (e.g., to wind and rain), improves harvest index (i.e., grain/straw ratio) and enhances biomass production [2]. Therefore, a wide range of studies have been done on the development of rice semi-dwarf mutants [3,6,7,8], which can be broadly categorized into phytohormone-dependent (e.g., brassinosteroids- or gibberellins-related) and phytohormone-independent mutants [6]. Previously, we reported a novel phytohormone-independent semi-dwarf rice mutant derived from the insertion of a single copy of transposon *Dissociator (Ds)* into the gene *OsCYP96B4* (*Oryza sativa CytochromeP450 96B4*), which resulted in defects in cell elongation and pollen germination [5]. As one of the largest protein-encoding gene families in plants, the cytochrome P450 (CYP) superfamily plays important roles in plant growth, development, and responses to biotic and abiotic stresses [9]. CYP96B subfamily belongs to the CYP96 family of the CYP86 clan. Recently, the functions of the CYP96B subfamily have been gradually revealed. CYP96B5 hydroxylates alkanes to primary alcohols and is involved in rice leaf cuticular wax synthesis [10]. *OsCYP96B4* is involved in secondary cell wall formation in rice [11], associated with the growth and drought stress responses in rice [12], and may be an important regulator of plant growth that affects plant height in rice [6]. However, to the best of our knowledge, currently, the systems metabolic effect of the *OsCYP96B4* gene mutation in rice is unclear, which is important for understanding the gene function and developing new semi-dwarf mutants.

Metabolomics is useful in characterizing the systems metabolic changes of biological systems to genetic modification or environmental stimuli [13,14,15,16]. Nuclear magnetic resonance (NMR) and mass spectrometry (MS) are the two dominant high-throughput analytical platforms employed in metabolomics, possessing their own advantages and providing complementary metabolic information [17]. For NMR analysis, it shows very high reproducibility, requires minimal sample preparation, detects in a non-selective manner and favors unambiguous metabolite identification [17]. Metabolomics has been widely applied in evaluating the metabolic response of plants to gene manipulation and biotic/abiotic stresses, e.g., the insertion of a moss Na^+^ transporter gene in rice and barley [18], the influence of *SUB1A* gene during submergence stress in rice [19], *Tri5* gene deletion in *Fusarium graminearum* [20], antisense thioredoxin s (*anti-trx-s*) introduction to wheat for pre-harvest sprouting resistance [21], *Fusarium oxysporum* inoculation in chickpea roots [22], brown planthopper infestation in pest-susceptible and -resistant rice plants [23]. Metabolomics has also been used to characterize the metabolic phenotypes of plant dwarf and semi-dwarf mutants, e.g., BW312 barley (*Hordeum vulgare*) semi-dwarf mutant [24], dwarf banana (*Musa* spp.; Musaceae) variants [25], tomato (*Solanum lycopersicum* L.) dwarf cultivar Micro-Tom [26], a dwarf genotype of soybean named MiniMax [27], and dwarfed *tcd2* (*totally cyanide deficient 2*) mutants of *Sorghum bicolor* (L.) Moench [28]. Such studies indicate that there are significant metabolic profile alterations in the dwarf/semi-dwarf mutant plants. However, to the best of our knowledge, currently, there is no metabolomics study on the dwarf rice.

In the present study, we investigated the differences in the metabolome and related gene expression levels among the *oscyp96b4* semi-dwarf mutant (M), ectopic expression (ECE), and wild-type (WT) rice using NMR-based metabolomics and quantitative real-time polymerase chain reaction (qRT-PCR). We aimed to (1) characterize the metabolic phenotypes of the mutant rice and (2) elucidate the systems metabolic effect of the mutation by integrating the metabolomics and gene expression data. The analysis provides essential information on the *OsCYP96B4* gene function and may serve as reference data for the development of new semi-dwarf mutants.

## 2. Results

### 2.1. Metabolite Profiling in Wild-Type and Mutant Rice

**Figure 1:**
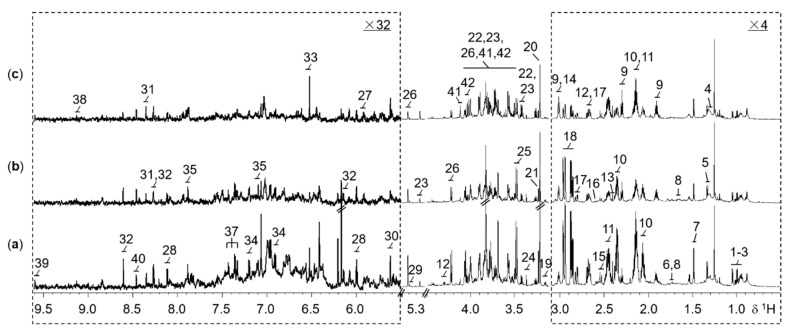
Representative 800 MHz ^1^H NMR spectra for the extracts of 2-week-old seedling shoots (without roots) from (**a**) the wild type, (**b**) the *oscyp96b4* semi-dwarf mutant, and (**c**) the *OsCYP96B4* ectopic expression rice lines. The dotted regions δ0.5−3.1 and δ5.5−9.61 were vertically expanded 4 and 32 times respectively, compared to the region δ3.1-4.5. Metabolite keys: 1, isoleucine; 2, leucine; 3, valine; 4, lipid; 5, threonine; 6, lysine; 7, alanine; 8, arginine; 9, γ-aminobutyrate; 10, glutamate; 11, glutamine; 12, malate; 13, succinate; 14, 2-oxoglutarate; 15, citrate; 16, methylamine; 17, aspartate; 18, asparagine; 19, ethanolamine; 20, choline; 21, phosphocholine; 22, β-glucose; 23, α-glucose; 24, methanol; 25, mono-methyl phosphate; 26, sucrose; 27, uridine; 28, uridine 5′-monophosphate (UMP); 29, allantoin; 30, uridine diphosphate glucuronic acid (UDP glucuronate); 31, adenosine; 32, adenosine monophosphate (AMP); 33, fumarate; 34, tyrosine; 35, histidine; 36, tryptophan; 37, phenylalanine; 38, N-methylnicotinate (trigonelline, NMNA); 39, nicotinamide mononucleotide (nicotinamide ribotide, NMN); 40, formate; 41, β-D-fructopyranose; 42, β-D-fructofuranose.

In the ^1^H NMR spectra of rice extracts, a total of 42 abundant metabolites were assigned (Table S1) according to 2D NMR experiments and literature reports [29]. Typical 1D ^1^H NMR spectra of WT, M, and ECE rice extracts were shown in Figure 1. The NMR spectra contain signals mainly from amino acids and derivatives, choline metabolism-related metabolites, carbohydrate metabolites, tricarboxylic acid (TCA) cycle intermediates, nucleotide metabolites, N-methylnicotinate, nicotinamide mononucleotide, lipid, formate, methylamine, mono-methyl phosphate, and methanol.

### 2.2. OsCYP96B4 Gene Mutation Induced Metabolic Changes in Mutant Rice

**Figure 2:**
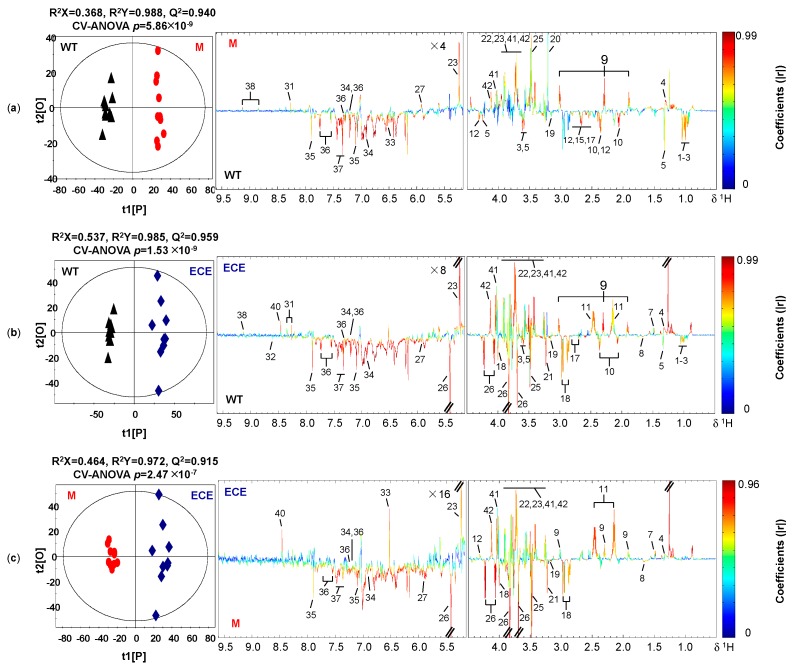
Cross-validated OPLS-DA scores plots (**left**) and the corresponding loadings plots (**right**) derived from the comparison of ^1^H NMR spectra for (**a**) the wild type (WT, ▲) and the *oscyp96b4* semi-dwarf mutant (M, ●), (**b**) the wild type (WT, ▲) and the *OsCYP96B4* ectopic expression (ECE, ◆), (**c**) the *oscyp96b4* semi-dwarf mutant (M, ●) and the *OsCYP96B4* ectopic expression (ECE, ◆). Metabolite keys are shown in Figure 1 and Table S1.

**Figure 3:**
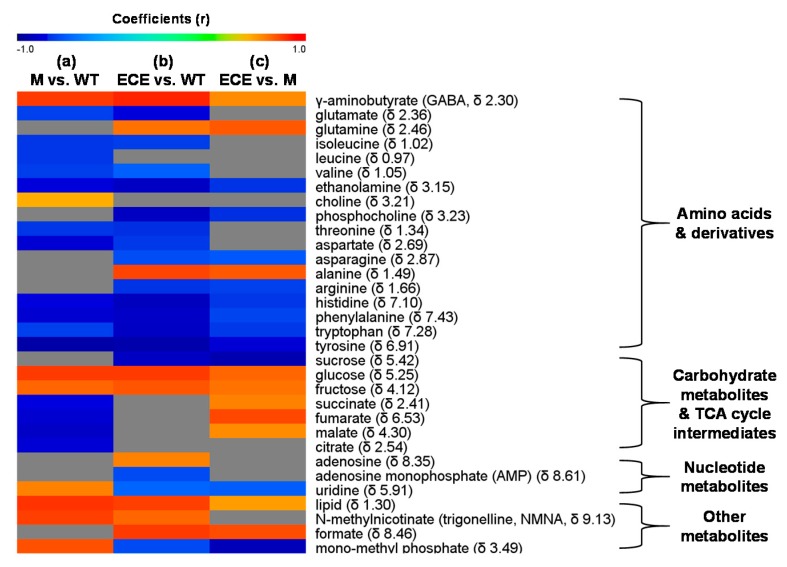
Heat map showing metabolites with significant level changes (*p* < 0.05) for (**a**) the *oscyp96b4* semi-dwarf mutant (M) vs. the wild type (WT), (**b**) the *OsCYP96B4* ectopic expression (ECE) vs. the wild type (WT), and (**c**) the *OsCYP96B4* ectopic expression (ECE) vs. the *oscyp96b4* semi-dwarf mutant (M). It was color-coded with the Pearson correlation coefficients from the corresponding OPLS-DA models, where a warm color (e.g., red) indicates significant increase of metabolites in M (**a**) or ECE (**b**,**c**) as compared to the counterpart, a cool color (e.g., blue), indicating significant decrease, and the grey color indicates no significant difference.

Clear differentiation in the metabolic profiles among the three rice phenotypes was shown in the PCA scores plots (Figure S1) and the corresponding OPLS-DA scores plots (Figure 2). The OPLS-DA model quality was evaluated by the sevenfold internal cross-validation, with R^2^(X) = 0.368, R^2^(Y) = 0.988, Q^2^ = 0.940 for the comparison between WT and M (Figure 2a), R^2^(X) = 0.537, R^2^(Y) = 0.985, Q^2^ = 0.959 for the comparison between WT and ECE (Figure 2b), and R^2^(X) = 0.464, R^2^(Y) = 0.972, Q^2^ = 0.915 for the comparison between M and ECE (Figure 2c). The model validities were demonstrated by CV-ANOVA of OPLS-DA models (Figure 2) and permutation tests on the corresponding PLS-DA models (Figure S2). Detailed metabolic profile difference among the three phenotypes were shown in the color-coded loadings plots of OPLS-DA models (Figure 2) and further summarized in Figure 3 and Table S2.

Compared to WT, M showed higher levels of γ-aminobutyrate, choline, carbohydrates (glucose, fructose), uridine, N-methylnicotinate, lipid, and mono-methyl phosphate, but lower levels of most amino acids (isoleucine, leucine, valine, threonine, glutamate, aspartate, tyrosine, histidine, tryptophan, phenylalanine), ethanolamine, and TCA cycle intermediates (malate, succinate, citrate, and fumarate) (*p* < 0.05, Figure 2a and Figure 3a, Table S2). ECE presented more alanine, γ-aminobutyrate, glutamine, carbohydrates (glucose, fructose), adenosine, N-methylnicotinate, lipid, and formate, but less amino acids (isoleucine, valine, threonine, arginine, glutamate, aspartate, asparagine, tyrosine, histidine, tryptophan, phenylalanine), choline metabolites (ethanolamine, phosphocholine), sucrose, nucleotide metabolites (uridine, adenosine monophosphate), and mono-methyl phosphate than WT (*p* < 0.05, Figure 2b and Figure 3b, Table S2). Compared with M, there were increases of γ-aminobutyrate, glutamine, alanine, carbohydrates (glucose, fructose), TCA cycle intermediates (succinate, fumarate and malate), lipid, and formate, but decreases of amino acids (asparagine, arginine, histidine, phenylalanine, tyrosine, tryptophan), choline metabolites (ethanolamine, phosphocholine), sucrose, uridine, and mono-methyl phosphate in ECE (*p* < 0.05, Figure 2c and Figure 3c, Table S2).

### 2.3. Gene Expression Analysis

**Figure 4:**
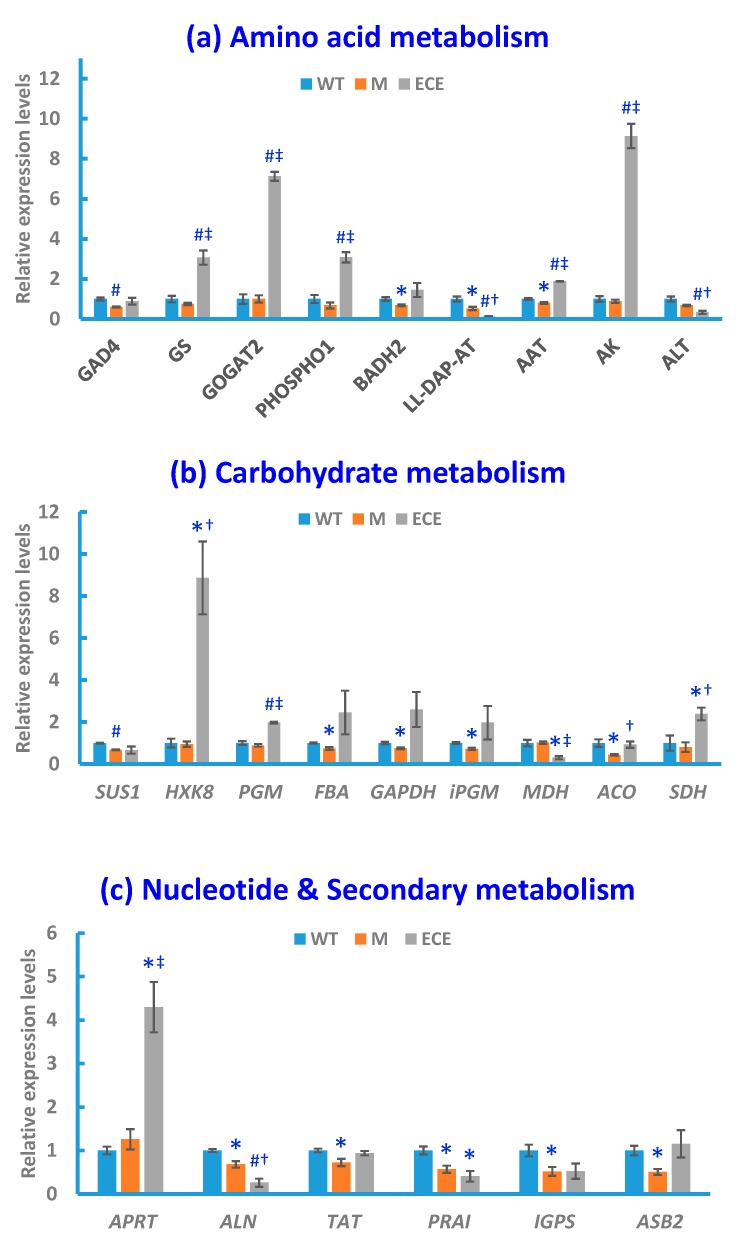
Gene expression levels in the wild-type (WT, blue bars), the *oscyp96b4* semi-dwarf mutant (M, orange bars), and the *OsCYP96B4* ectopic expression (ECE, grey bars) rice lines measured by qRT-PCR. Data shown are means ± SE of three biological replicates each with three technical replicates (* *p* < 0.05, ^#^*P* < 0.01, as compared to WT; † *p* < 0.05, ‡ *p* < 0.01, as compared to M).

The qRT-PCR analysis was performed to acquire supporting information for the aforementioned metabolic changes induced by *OsCYP96B4* gene mutation. Compared with WT, significant alterations (*p* < 0.05) in M or ECE were observed for the expression levels of key enzyme-encoding genes, responsible for the regulation of GABA shunt, glutamate/glutamine metabolism, choline metabolism, carbohydrate metabolism, nucleotide metabolism, and secondary metabolism (Figure 4).

Along with the metabolite level changes, *OsCYP96B4* gene mutation induced significant alterations in the expression levels of key genes in GABA shunt and glutamate/glutamine metabolism, including the down-regulation of glutamate decarboxylase 4 gene (*GAD4*) in M and the up-regulation of glutamate synthase 2 gene (*GOGAT2*) and glutamine synthetase gene (*GS*) in ECE. *OsCYP96B4* gene mutation also resulted in significant changes of gene expression related to choline metabolism, i.e., down-regulation of betaine aldehyde dehydrogenase 2 gene (*BADH2*) in M and up-regulation of phosphoethanolamine/phosphocholine phosphatase gene (*PHOSPHO1*) in ECE. In addition, significant alterations were also observed for the gene expression pertaining to the metabolism of other amino acids, including the down-regulation of aspartate aminotransferase gene (*AAT*) and LL-diaminopimelate aminotransferase gene (*LL-DAP-AT*) in M, along with the up-regulation of *AAT* and aspartate kinase gene (*AK*) and down-regulation of alanine transaminase gene (*ALT*) and *LL-DAP-AT* in ECE (Figure 4a).

There were significant changes in the gene expression involved in carbohydrate metabolism, including significant down-regulation of genes encoding sucrose synthase 1 (*SUS1*), fructose-bisphosphate aldolase (*FBA*), glyceraldehyde 3-phosphate dehydrogenase (*GAPDH*), 2,3-bisphosphoglycerate-independent phosphoglycerate mutase (*iPGM*) and aconitase (*ACO*) in M, along with the up-regulation of genes encoding hexokinase-8 (*HXK8*), phosphoglucomutase (*PGM*), and succinate dehydrogenase (*SDH*) and down-regulation of genes encoding malate dehydrogenase (*MDH*) in ECE (Figure 4b).

Significant changes also occurred in the gene expression related to nucleotide metabolism, i.e., the up-regulation of adenine phosphoribosyltransferase gene (*APRT*) in ECE, and down-regulation of allantoinase (*ALN*) gene in both M and ECE (Figure 4c). In addition to the significant alterations of gene expression in primary metabolism, *OsCYP96B4* gene mutation also led to significant changes of gene expression in secondary metabolism, i.e., the down-regulation of tyrosine aminotransferase gene (*TAT*), phosphoribosylanthranilate isomerase gene (*PRAI*), indole-3-glycerol phosphate synthase gene (*IGPS*), anthranilate synthase beta subunit 2 gene (*ASB2*) in M, and the down-regulation of *PRAI* in ECE (Figure 4c).

For the comparison between ECE and M, the gene expression alterations were nearly the same as the comparison between ECE and WT, except for the significant increase of *ACO* and no significant change of *PRAI* in ECE when compared with M (at the significance level of 0.05, Figure 4a–c).

## 3. Discussion

**Figure 5:**
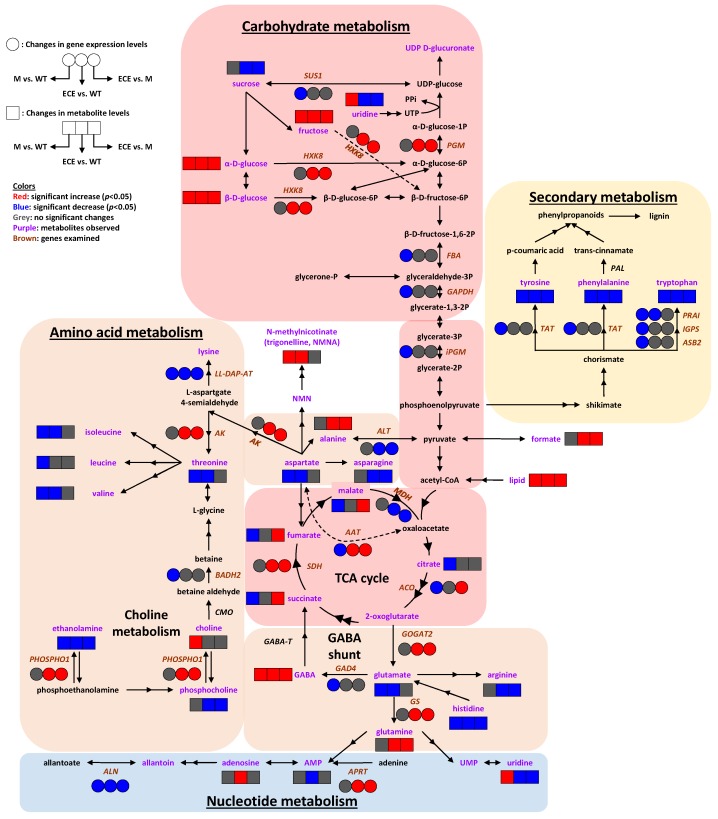
Systems metabolic reprogramming induced by *OsCYP96B4* gene mutation in rice. Symbols: boxes (☐) represent metabolite levels; circles (◯) represent gene expression levels; both the solid and dashed arrows indicate the enzyme catalyzed metabolic reactions, where the dashed arrows were intentionally used to prevent the intersection between solid ones. Colors: red indicates significant increase (*p* < 0.05), blue indicates significant decrease (*p* < 0.05), grey indicates no significant change; purple letters denote identified metabolites; italic brown letters denote genes with examined transcript levels. Metabolite abbreviations: AMP, adenosine monophosphate; GABA, γ-aminobutyrate; NMN, nicotinamide mononucleotide; NMNA, N-methylnicotinate; PPi, diphosphate; UDP, uridine diphosphate; UMP, uridine 5’-monophosphate; UTP, uridine triphosphate. Gene abbreviations: *SUS1*, sucrose synthase 1; *PGM*, phosphoglucomutase; *HXK8*, hexokinase-8; *FBA*, fructose-bisphosphate aldolase; *GAPDH*, glyceraldehyde 3-phosphate dehydrogenase; *iPGM*, 2,3-bisphosphoglycerate-independent phosphoglycerate mutase; *SDH*, succinate dehydrogenase; *MDH*, malate dehydrogenase; *ACO*, aconitase; *PAL*, phenylalanine ammonia-lyase; *TAT*, tyrosine aminotransferase; *PRAI*, phosphoribosylanthranilate isomerase; *IGPS*, indole-3-glycerol phosphate synthase; *ASB2*, anthranilate synthase beta subunit 2; *LL-DAP-AT*, LL-diaminopimelate aminotransferase; *AK*, aspartate kinase; *ALT*, alanine transaminase; *AAT*, aspartate aminotransferase; *BADH2*, betaine aldehyde dehydrogenase 2; *CMO*, choline monooxygenase; *PHOSPHO1*, phosphoethanolamine/phosphocholine phosphatase; *GABA-T*, GABA transaminase; *GAD4*, glutamate decarboxylase 4; *GOGAT2*, glutamine:2-oxoglutarate amidotransferase 2; *GS*, glutamine synthetase; *APRT*, adenine phosphoribosyltransferase; *ALN*, allantoinase.

The aforementioned metabolomics and gene expression data showed that *OsCYP96B4* gene mutation resulted in comprehensive metabolic responses in rice plants. The responses were summarized in Figure 5, mainly including alterations in amino acid metabolism, carbohydrate metabolism, nucleotide metabolism, and secondary metabolism. In general, such changes were more comprehensive in ECE than in M when compared with WT, especially on the gene expression levels. Moreover, significant differences were observed in the metabolic and gene expression levels between ECE and M. These differences may be related to the developmental disparities in the two mutants. The *oscyp96b4* mutant displayed a semi-dwarf phenotype, but with the development of panicles and seeds (i.e., complete mature plant formation). However, the ECE plants remained severely dwarf, without any panicle formation, and normally died after about 4 weeks in the pot. Taken together, the results shed light on the *OsCYP96B4* gene function and may be associated with the plant phenotype (i.e., dwarfism).

### 3.1. Amino Acid Metabolism

The GABA shunt is comprised of three steps, including the α-decarboxylation of glutamate to GABA by glutamate decarboxylase (*GAD*), the conversion of GABA to succinic semialdehyde by GABA transaminase (*GABA-T*), and the oxidization of succinic semialdehyde to succinate catalyzed by succinic semialdehyde dehydrogenase (*SSADH*) [30,31]. There are reported connections between GABA shunt and plant dwarfism. The suppression of *GABA-T* induces prominent GABA accumulation, dwarfism, and infertility in the tomato [32]. Rice plants overexpressing *OsGAD2ΔC* have dwarf phenotypes [33]. In the present study, there was significant increase of GABA and decrease of glutamate levels in both M and ECE. Besides, the succinate level and the *GAD4* gene expression was down-regulated in M. Although both glutamate and *GAD4* were down-regulated in M, other metabolic pathways (e.g., arginine and proline metabolism) may contribute to the increase of GABA level. It indicates that *OsCYP96B4* functions affect rice GABA shunt, which may be connected with the dwarfism phenotype [32,33].

The glutamine synthetase/glutamine:2-oxoglutarate amidotransferase (i.e., GS/GOGAT) cycle plays a regulatory role in the nitrogen assimilation process in plants [34]. While glutamine synthetase (*GS*) catalyzes the conversion of glutamate and ammonia into glutamine, glutamate synthase (i.e., *GOGAT*, or glutamine:2-oxoglutarate amidotransferase) catalyzes the formation of glutamate from glutamine and 2-oxoglutarate. Here, the significant decrease of glutamate was observed in both M and ECE, together with the up-regulated *GS*, *GOGAT2* gene expression, and glutamine level in ECE. It suggests the function of *OsCYP96B4* on rice glutamate and glutamine metabolism, along with GS/GOGAT cycle, which may be connected with the nitrogen assimilation process [34] and the dwarfism phenotype.

Isoleucine, leucine, and valine are three branched-chain amino acids (BCAAs). Here, significant decrease of isoleucine and valine were observed in both M and ECE, along with the significantly reduced leucine level in M. As both valine and isoleucine possess the glucogenic property, their level decrease and the increase of glucose concentration may indicate the possible gluconeogenesis process in the mutant rice. Although at present there are no relevant reports, it suggests the effect of *OsCYP96B4* mutation on rice branched-chain amino acid metabolism, which may be connected with the dwarfism phenotype.

Phosphoethanolamine/phosphocholine phosphatase (*PHOSPHO1*) catalyzes the conversion of phosphoethanolamine to ethanolamine and the conversion of phosphocholine to choline. Choline can be further converted to betaine by the action of choline monooxygenase (*CMO*) and betaine aldehyde dehydrogenase 2 (*BADH2*). It was shown that simultaneous expression of *Spinacia oleracea* chloroplast *CMO* and *BADH* genes contribute to dwarfism in transgenic *Lolium perenne* [35]. In the current study, there were significantly decreased ethanolamine level and *BADH2* gene expression level, along with significantly increased choline level in M. While, there were significantly decreased ethanolamine and phosphocholine levels and significantly increased *PHOSPHO1* gene expression level in ECE. These observations suggest the influence of *OsCYP96B4* function on rice choline metabolism and dwarfism [35].

In addition, *OsCYP96B4* mutation showed potential effects on the metabolism of other amino acids, including the significant decrease of threonine, aspartate, histidine levels, and *LL-DAP-AT* gene expression in both M and ECE, the down-regulation of *AAT* expression level in M, and the significantly decreased levels of asparagine, arginine, and *ALT* expression along with the significantly increased levels of alanine and *AK* expression in ECE.

### 3.2. Carbohydrate Metabolism

Sucrose synthase (*SUS*) is a key enzyme for the regulation of carbon partitioning in plants by providing UDP-glucose as a substrate for the biosynthesis of cellulose and other polysaccharides [36]. It has been shown that AtCesA8::SUS3 transgenic rice plants exhibited largely improved biomass saccharification and lodging resistance by reducing cellulose crystallinity and increasing cell wall thickness [36]. *OsIDD2* overexpression leads to severely dwarfed rice plants and *OsIDD2* negatively regulates the transcription of genes involved in lignin biosynthesis, cinnamyl alcohol dehydrogenase 2 and 3 (*CAD2* and *3*), and sucrose metabolism sucrose synthase 5 (*SUS5*) [37]. In the present study, although there were significant increases of glucose, fructose, and uridine levels in M, the significant decrease of *SUS1* gene expression may lead to the unchanged level of sucrose. While for ECE, the significantly increased levels of glucose, fructose, and *HXK8* and *PGM* gene expression and the decreased uridine level may result in the decrease of sucrose level, in spite of the unchanged *SUS1* gene expression. It suggests the influence of *OsCYP96B4* on sucrose metabolism, which may be connected with dwarfism [37]. Besides, the significantly decreased *FBA*, *GAPDH*, and *iPGM* gene expression levels in M further supported the effect of *OsCYP96B4* gene mutation on rice carbohydrate metabolism.

The TCA cycle is carried out through a variety of interconnected enzymatic reactions, e.g., the conversion of succinate to fumarate by succinate dehydrogenase (*SDH*), the oxidation of malate to oxaloacetate via malate dehydrogenase (*MDH*), and the transformation of citrate to *cis*-aconitate and then isocitrate by aconitase (*ACO*). *OsAPX2* (rice ascorbate peroxidase 2) knock-out leads to shoot dwarfing and up-regulation of enzymes linked to glycolysis and TCA cycle in rice flag leaves [38]. *Arabidopsis thaliana* mutants lacking plastidial NAD-dependent MDH (*pdnad-mdh*) are embryo-lethal, and constitutive silencing (*miR-mdh-1*) leads to a dwarfed phenotype [39]. In the present study, there were significant decreases of TCA cycle intermediates (i.e., succinate, fumarate, malate, and citrate) and *ACO* gene expression level in M, along with the significant up-regulation of *SDH* and the down-regulation of *MDH* gene expression levels in ECE. It is inferred that the perturbation in TCA cycle is related to *OsCYP96B4* gene function and may be connected with the rice dwarfism phenotype [38,39].

### 3.3. Nucleotide Metabolism

As an important intermediate in the purine metabolism, adenosine monophosphate (AMP) is formed from adenine by adenine phosphoribosyltransferase (*APRT*) [40] and is interconvertible with adenosine through hydroxylation and phosphorylation reactions. Allantoin, an intermediary metabolite of purine catabolism, is hydrolyzed to allantoate under the catalysis of allantoinase (*ALN*) [41]. Similarly, uridine and UMP are two interconvertible intermediates in the pyrimidine metabolism. *APRT* was shown to be potentially involved in thermo-sensitive genic male sterility (TGMS) in the rice line ‘Annong S-1′ [42] and associated with growth retardation and male sterility in Arabidopsis [43]. In the current study, there was significant increase of adenosine and *APRT* gene expression, but significant decrease of AMP, uridine, and *ALN* gene expression level in ECE, along with significant increase of uridine and the significant decrease of *ALN* gene expression level in M. It suggests the potential function of *OsCYP96B4* on rice nucleotide metabolism, which may be associated with the rice dwarfism phenotype. However, to the best of our knowledge, currently, there are no reports on such association.

### 3.4. Secondary Metabolism

The shikimate and aromatic amino acids (AAA) biosynthesis pathways represent a link between primary and secondary metabolism in plants [44]. The catabolism of AAAs results in a variety of secondary metabolites, e.g., phenylpropanoids and flavonoids from phenylalanine, tocochromanols and phenylpropanoids from tyrosine, and indole-containing metabolites from tryptophan [45]. Cytochrome P450 monooxygenases (*CYP450s*) play important roles in the biosynthesis of plant secondary metabolites, including phenylpropanoids, terpenes, and alkaloids [46]. As one of the major phenylpropanoid pathway end-products, lignin is a major component of the secondary cell wall and is vital for providing mechanical strength to reduce lodging stress in plants [47,48]. Phenylalanine ammonia-lyase (*PAL*) catalyzes the conversion of phenylalanine to *trans*-cinnamic acid and plays an important role in the biosynthetic pathway of lignin [49]. In the dwarf rice mutant Fukei 71, elevated levels of p-coumaric acid (PCA), ferulic acid (FA), and *PAL* were observed in the abnormal parenchyma tissue, indicating that the abnormal activation of phenylpropanoid pathway leads to the biosynthesis of polysaccharide-linked FA and PCA [50]. The AAAs metabolism is also connected with dwarfism. The overexpression of a rice tyrosine decarboxylase (*TyDC*) leads to tyramine accumulation in rice cells and causes a dwarf phenotype via reduced cell division [51]. The suppression of serotonin N-acetyltransferase 2 (*SNAT2*) leads to melatonin-deficient rice with a semidwarf phenotype [52]. Our present study showed that there was significant decrease in levels of phenylalanine, tyrosine, and tryptophan in both M and ECE, along with the significant decrease of *TAT*, *PRAI*, *IGPS*, and *ASB2* expression levels in M and the significant decrease of *PRAI* expression level in ECE. It may indicate the declined synthesis of phenylalanine, tyrosine, and tryptophan and reflect the function of *OsCYP96B4* on shikimate-mediated secondary metabolism in rice, which may be possibly connected with the dwarfism phenotype [51,52].

### 3.5. Other Metabolism

In our previous study [5], GC-MS lipid profiling was performed on non-polar extracts of rice leaves from WT and M. Three classes of lipid were analyzed, i.e., plant glycolipids, the common membrane phospholipid classes, and the minor membrane lipid metabolites. Compared with WT, there were no significant alteration in total monogalactosyldiacylglycerol (MGDG) or total digalactosyldiacylglycerol (DGDG), but significant increase of MGDG 34:6, and significant decreases of DGDG 34:1, DGDG 36:2, total phosphatidylglycerol (PG), PG 36:2, total lysoPG and lysoPG 16:1 in M. While, in the present study, untargeted NMR metabolic profiling was performed on the polar extraction of the whole 2-week-old seedling shoots without roots. The accumulation of total lipids, with a wider species coverage than our previous study [5], was observed in both M and ECE. Though the difference in plant material, extraction method, and lipid coverage may lead to different results, both studies suggested that *OsCYP96B4* might be involved in rice plant lipid metabolism. The accumulation of lipids observed here may indicate the reduced conversion of lipids to other metabolites or the increased synthesis of lipids from other metabolites. The significant elevation of N-methylnicotinate level in both M and ECE may indicate the potential effect of this mutation on vitamin B3 metabolism. In addition, the significant increase of formate concentration suggests altered formate metabolism in ECE.

## 4. Materials and Methods

### 4.1. Plant Materials

The *Japonica* rice (*Oryza sativa* ssp. *japonica* cv. Nipponbare) was used as the wild-type (WT) rice plant for the current study. The *oscyp96b4* semi-dwarf (M) was a *Ds* insertion mutant, and the *OsCYP96B4* ectopic expression (ECE) lines showing the most severe dwarf phenotype were generated constitutively expressing the *OsCYP96B4* gene in the Nipponbare background. The detailed descriptions about the mutant and ectopic expression lines can be found in our previous report [5]. For each genotype of rice plants (i.e., WT, M, and ECE, Figure S3), 19 seedlings were grown in a Phytatray™ II (Sigma-Aldrich, St. Louis, MO, USA) containing half strength Murashige and Skoog medium [53] (Sigma-Aldrich, St. Louis, MO, USA) in a plant growth room maintained at 28 °C with 12 h dark and 12 h light. For metabolites extraction, 10 whole 2-week-old seedling shoots without roots were used for each genotype. While for qRT-PCR, 3 independent biological replicates were used, where each replicate was made up of RNA extracted from 3 seedlings pooled together for better representation. Each plant sample was collected individually into a 2 mL Eppendorf tube, weighed, immediately snap-frozen in liquid nitrogen and stored at −80 °C until further analyses.

### 4.2. Metabolome Analysis

#### 4.2.1. Chemicals

Analytical grade methanol, K_2_HPO_4_·3H_2_O, NaH_2_PO_4_·2H_2_O and sodium azide (NaN_3_) were purchased from Sigma-Aldrich (St. Louis, MO, USA). Sodium 3-trimethylsilyl [2,2,3,3−^2^H_4_]-propionate (TSP) and deuterium oxide (D_2_O, 99.9% D) were purchased from Cambridge Isotope Laboratories, Inc. (Tewksbury, MA, USA). The phosphate buffer for NMR analysis was prepared by dissolving K_2_HPO_4_ and NaH_2_PO_4_ in water (0.15 M, pH 7.44, K_2_HPO_4_/NaH_2_PO_4_ = 4:1) containing 0.001% TSP, 0.1% NaN_3_, and 50% D_2_O [54].

#### 4.2.2. Plant Extraction and Sample Preparation

For each of the three groups (i.e., WT, M, and ECE), 10 biological replicates of rice plants were used for the extraction and subsequent NMR-based metabolomics analysis. Each rice plant was extracted individually using a method modified from previous reports [23,54]. Firstly, the rice sample-containing tube was snap-frozen in liquid nitrogen and the rice tissue was then swiftly ground into fine powder using a pre-cooled pestle. Pre-cooled methanol/water (v/v = 2/1, −20 °C) was added into the homogenized sample at a ratio of 600 µL per 100 mg powder. Afterwards, the mixture was further homogenized using a 2010 Geno/Grinder^®^ (SPEX Sample Prep, Metuchen, NJ, USA) at 1300 rpm for 90 s. Then the homogenate mixture was sonicated in an ice bath with 10 cycles of 30 s sonication and 30 s break. Following centrifugation (14,489× *g*, 10 min, 4 °C), the supernatant was collected, and the remaining pellets were further treated twice using the same procedure. Three supernatants were combined and lyophilized after removal of methanol *in vacuo* (CentriVap Centrifugal Vacuum Concentrators, Labconco, MO, USA). Each dried extract was reconstituted into 600 μL phosphate buffer prepared as previously described. After a final centrifugation, 500 μL supernatant was transferred into a 5 mm NMR tube for NMR analysis.

#### 4.2.3. NMR Spectroscopy

All NMR spectra were acquired on a Bruker Avance 800 MHz NMR spectrometer (800.15 MHz for proton frequency) at 302 K using a 5 mm cryoprobe (Bruker Biospin, Rheinstetten, Germany) [55]. A one-dimensional (1D) ^1^H NMR spectrum was acquired for each sample in a random order using the first increment of the gradient selected NOESY pulse sequence (recycle delay−*G*_1_−90°−*t*_1_−90°−*t*_m_−*G*_2_−90°−acquisition) with water presaturation during both the recycle delay (2 s) and mixing time (t_m_, 80 ms) [56]. For each spectrum, a total of 64 transients were collected into 32,768 data points over a spectral width of 16,025 Hz with a 90° pulse length adjusted to around 11 μs. For selected samples, a variety of two-dimensional (2D) NMR spectra [54] were acquired for the purpose of resonance assignment, including ^1^H−^1^H Correlation Spectroscopy (COSY), ^1^H−^1^H Total Correlation Spectroscopy (TOCSY), ^1^H J-Resolved Spectroscopy (JRES), ^1^H−^13^C Heteronuclear Single Quantum Correlation Spectroscopy (HSQC), and ^1^H−^13^C Heteronuclear Multiple Bond Correlation Spectroscopy (HMBC).

After Fourier transformation with 1 Hz exponential line broadening and 65,536 data points zero-filling, each 1D spectrum was manually corrected for phase and baseline distortions and referenced to TSP (δ 0.00) using Topspin 2.0 (Bruker Biospin, Germany). The spectra regions δ 0.500−9.610 were segmented into discrete bins of 0.003 ppm width using AMIX (V3.9.15, Bruker Biospin, Germany) [57]. Spectra regions with imperfect water suppression (δ 4.500−5.170) or residual methanol signal (δ 3.356−3.370) were discarded. The intersample chemical-shift variations for some metabolites were manually corrected to prevent the possible adverse effect on subsequent data analysis [58].

#### 4.2.4. Multivariate Data Analysis

The integral of each included bin was normalized to the summed integral of all included bins. The normalized data was then utilized for multivariate data analysis using SIMCA-P^+^ (V11.0 and 13.0, Umetrics AB, Umea, Sweden). Initially, for the overview of data distribution and detection of possible outliers, Principal Component Analysis (PCA) was performed on mean-centered data using two principal components. Then, Projection to Latent Structure Discriminant Analysis (PLS-DA) and Orthogonal Projection to Latent Structure Discriminant Analysis (OPLS-DA) were performed on unit-variance scaled data by using grouping information as Y-matrix [59]. Two PLS components were calculated for PLS-DA models, while one PLS and one orthogonal component were used for OPLS-DA models. Both supervised models were validated using a 7-fold cross-validation method [59]. Further assessments of model quality were also performed, including a permutation test with 200 permutations for PLS models [60] and ANOVA of the cross-validated residuals (CV-ANOVA) tests for OPLS-DA models [61].

The OPLS-DA models were interpreted as back-transformed and color-coded correlation coefficients loadings plots [62] (MATLAB 7.0, The Mathworks Inc., Natick, MA, USA), where the colors indicate the significance of differentiating metabolites, with a warm color (e.g., red) being more significant than a cool color (e.g., blue). The cutoffs for correlation coefficients were chosen on the basis of discrimination significance (*p* < 0.05), e.g., a cutoff value (|r|) of 0.602 was corresponding to the sample number (n) of 10. Differentiating metabolites were also summarized in a heat map, color-coded with the Pearson correlation coefficients from the OPLS-DA models (MeV version 4.9.0).

### 4.3. RNA Extraction and qRT-PCR Analysis

For RNA extraction and subsequent qRT-PCR analysis, 3 biological replicates were used for each of the three genotypes (i.e., WT, M, and ECE). Each replicate consisted of 3 seedlings pooled together before RNA extraction, and the seedlings were from the same batch used for the metabolomics analysis. Moreover, each qRT-PCR reaction was performed with 3 technical replicates. Total RNA was extracted using Qiagen RNeasy Mini Kit (Cat No. 74904) from 2-week-old seedling shoots without roots according to the manufacturer’s instructions. The RNA quality was determined by a Nanodrop ND-1000 spectrophotometer (Nanodrop Technologies), and RNA samples with A260/A280 ratios between 1.9 and 2.1 were selected for further analysis. One microgram total RNA was reverse transcribed using Maxima First-Strand cDNA Synthesis Kit (ThermoFisher Scientific, Waltham, MA, USA, Cat No. K1671), as per the manufacturer’s instructions. The qRT-PCR analysis was carried out using selected gene-specific primer pairs (Table S3) on *BIO-RAD* CFX384 Real-Time system with denaturation at 95 °C for 10 min, followed by 40 cycles of denaturation at 95 °C for 15s and annealing/extension at 60 °C for 1 min. The amplification of an *ACTIN* gene (*OsACT1*) was used as an internal control to normalize the data. Melting curve analyses were performed to confirm the amplicon specificity [63]. The values were expressed as the average of three independent biological samples, each averaged from its three technical replicates. The relative expression levels were calculated using the 2^−∆∆Ct^ method [64]. Differentially expressed transcripts were derived from two-sided unpaired t-tests, with a *p* value of less than 0.05 considered to be statistically significant. The data analysis and charting (with reference to WT) was performed using Microsoft Excel 2016 (Microsoft, Redmond, WA, USA).

### 4.4. Metabolic Pathway Analysis

Based on the differential metabolites and transcripts, the comprehensive metabolic responses in rice plants induced by *OsCYP96B4* gene mutation were mapped onto relevant metabolic pathways, with reference to the KEGG pathway database [65].

## 5. Conclusions

In the present study, the combination of NMR-based metabolomics and qRT-PCR analyses revealed that there were systems alteration in the metabolic phenotypes of semi-dwarf mutant (M) and ectopic expression (ECE) rice lines in comparison with the wild-type (WT) rice, as a result of the *OsCYP96B4* gene mutation. Such changes included the significant effect on amino acid metabolism (e.g., GABA shunt, glutamate and glutamine metabolism, branched-chain amino acid metabolism, choline metabolism), carbohydrate metabolism (e.g., sucrose metabolism, TCA cycle), nucleotide metabolism, and shikimate-mediated secondary metabolism. The present findings provide useful information on understanding the *OsCYP96B4* gene function possibly pertaining to the metabolism and dwarfism, which may be helpful for the development of valuable new semi-dwarf plant mutants in the future.

## Supplementary Materials

The following are available online at https://www.mdpi.com/1422-0067/21/6/1924/s1, Figure S1: PCA scores plots derived from ^1^H NMR spectra of rice plant extracts from different groups; Figure S2: Permutation test results (with 200 permutations) for PLS-DA models (with 2 components) derived from ^1^H NMR spectra of rice plant extracts from different groups; Figure S3: Representative phenotypes of the 2-week-old (A) *oscyp96b4* semi-dwarf mutant (M), (B) *OsCYP96B4* ectopic expression (ECE), and (C) wild-type (WT) rice plants; Table S1: ^1^H and ^13^C NMR assignment for metabolites in rice plant extracts; Table S2: OPLS-DA loadings correlation coefficients; Table S3: Primers for quantitative real-time PCR analysis on selected genes.

## Abbreviations

Ds	Dissociator
ECE	Ectopic expression
GABA	γ-aminobutyrate
M	Mutant
NMR	Nuclear magnetic resonance
OPLS-DA	Orthogonal projection to latent structure discriminant analysis
OsCYP96B4	Oryza sativa Cytochrome P450 96B4
PCA	Principal component analysis
PLS-DA	Projection to latent structure discriminant analysis
qRT-PCR	Quantitative real-time polymerase chain reaction
TCA	Tricarboxylic acid
WT	Wild type
