# Supplementary material for: Systems Metabolic Alteration in a Semi-Dwarf Rice Mutant Induced by OsCYP96B4 Gene Mutation
Source: Int J Mol Sci. 2020 Mar 11;21(6):1924. doi: 10.3390/ijms21061924 (PMC7139402; doi:10.3390/ijms21061924)
Supplement: Supplementary file 1 [file ijms-21-01924-s001.pdf]

Supporting Information:

Table 1. ^1H and ^{13}C NMR assignment for metabolites in rice plant extracts.

No.	metabolites	moieties	δ^{1}H	δ^{13}C
1	isoleucine	δCH_3	0.94(t)[a]	11.8
		γ'CH_3	1.02(d)	15.3
		half γCH_2	1.27(m)	24.7
		half γCH_2	1.48(m)	24.7
		βCH	1.98(m)	36.0
		αCH	3.68(d)	60.0
2	leucine	δ'CH_3	0.96(d)	21.7
		δCH_3	0.97(d)	22.6
		γCH	1.70(m)	24.3
		βCH_2	1.74(m)	40.2
		αCH	3.74(t)	54.0
3	valine	γ'CH_3	1.00(d)	17.3
		γCH_3	1.05(d)	18.5
		βCH	2.28(m)	29.5
		αCH	3.62(d)	60.8
4	lipid	C**H**$_2$$CH_2$$CH_2$CO	1.30(m)	29.0
		C**H**$_2$$CH_2$CO	1.54(m)	26.4
		C**H**$_2$CO	2.17(m)	38.3
5	threonine	γCH_3	1.34(d)	20.2
		αCH	3.60(d)	61.1
		βCH	4.26(m)	66.6
6	lysine	γCH_2	1.46(m)	22.1
		δCH_2	1.73(m)	27.1
		βCH_2	1.90(m)	30.4
		εCH_2	3.03(t)	40.0
		αCH	3.76(t)	55.4
7	alanine	βCH_3	1.49(d)	16.8
		αCH	3.79(q)	51.2
8	arginine	half γCH_2	1.66(m)	24.6
		half γCH_2	1.74(m)	24.6
		βCH_2	1.93(m)	28.3
		δCH_2	3.25(t)	41.1
		αCH	3.77(#)	55.2
9	γ-aminobutyrate	βCH_2	1.91(qu)	24.4
		αCH_2	2.30(t)	35.0

No.	metabolites	moieties	δ¹H	δ¹³C
		γCH_2	3.02(t)	40.0
10	glutamate	half βCH_2	2.07(m)	27.7
		half βCH_2	2.14(m)	27.7
		γCH_2	2.36(m)	34.2
		αCH	3.77(t)	55.3
11	glutamine	βCH_2	2.14(m)	27.1
		γCH_2	2.46(m)	31.6
		αCH	3.78(t)	54.7
12	malate	half CH_2	2.38(dd)	43.3
		half CH_2	2.68(dd)	43.3
		CH	4.30(dd)	71.0
13	succinate	CH	2.41(s)	34.7
14	2-oxoglutarate	βCH_2	2.45(t)	#
		γCH_2	3.01(t)	#
15	citrate	half CH_2	2.54(d)	46.0
		half CH_2	2.67(d)	46.0
16	methylamine	CH_3	2.61(s)	25.6
17	aspartate	half βCH_2	2.69(dd)	37.3
		half βCH_2	2.82(dd)	37.3
		αCH	3.90(dd)	52.5
18	asparagine	half βCH_2	2.87(dd)	35.3
		half βCH_2	2.96(dd)	35.3
		αCH	4.01(dd)	51.9
19	ethanolamine	C**H**$_2$$NH_2$	3.15(t)	42.1
		C**H**$_2$OH	3.84(t)	58.1
20	choline	$N(CH_3)_3$	3.21(s)	54.5
		NCH_2	3.53(m)	68.1
		OCH_2	4.07(m)	56.3
21	phosphocholine	$N(CH_3)_3$	3.23(s)	54.7
		NCH_2	3.60(m)	67.1
		OCH_2	4.18(m)	58.8
22	β-glucose	1-CH	4.65(d)	96.6
		2-CH	3.26(dd)	74.8
		3-CH	3.50(t)	76.4
		4-CH	3.41(dd)	70.2
		5-CH	3.47(#)	76.6
		half 6-CH_2	3.73(dd)	61.4
		half 6-CH_2	3.91(dd)	61.4
23	α-glucose	1-CH	5.25(d)	92.7
		2-CH	3.55(dd)	72.0

No.	metabolites	moieties	δ¹H	δ¹³C
		3-CH	3.72(dd)	73.4
		4-CH	3.43(dd)	70.2
		5-CH	3.83(m)	72.7
		half 6-CH_2	3.77(#)	61.1
		half 6-CH_2	3.85(#)	61.1
24	methanol	CH_3	3.36(s)	49.6
25	mono-methyl phosphate	CH_3	3.49(d)	52.2
26	sucrose	1-CH (fructose)	3.69(d)	61.9
		6-CH (fructose)	3.83(m)	63.1
		5-CH (fructose)	3.91(m)	82.0
		4-CH (fructose)	4.06(t)	74.7
		3-CH (fructose)	4.23(d)	77.1
		2-C (fructose)		104.3
		4-CH (glucose)	3.49(dd)	69.8
		2-CH (glucose)	3.57(dd)	71.7
		3-CH (glucose)	3.78(t)	73.1
		6-CH (glucose)	3.83(m)	60.8
		5-CH (glucose)	3.85(m)	73.0
		1-CH (glucose)	5.42(d)	92.9
27	uridine	2-CH (ribose)	4.35(dd)	#
		5-CH (ring)	5.91(d)	#
		1-CH (ribose)	5.92(d)	#
		6-CH (ring)	7.87(d)	#
28	uridine 5′-monophosphate (UMP)	2-CH (ribose)	4.41(#)	#
		5-CH (ring)	5.99(d)	#
		1-CH (ribose)	6.00(d)	#
		6-CH (ring)	8.10(d)	#
29	allantoin	CH	5.39	63.9
30	uridine diphosphate glucuronic acid (UDP glucuronate)		5.62(t)	108.0
31	adenosine	3-CH (ribose)	4.45(#)	#
		1-CH (ribose)	6.08(d)	#
		8-CH (ring)	8.26(s)	#
		2-CH (ring)	8.35(s)	#
32	adenosine monophosphate (AMP)	3-CH (ribose)	4.53(#)	#
		1-CH (ribose)	6.14(d)	87.5
		8-CH (ring)	8.27(s)	153.2
		2-CH (ring)	8.61(s)	140.5
33	fumarate	CH	6.53(s)	#
34	tyrosine	3,5-CH (ring)	6.91(d)	116.4
		2,6-CH (ring)	7.20(d)	131.5
		4-C (ring)		155.8

No.	metabolites	moieties	δ¹H	δ¹³C
35	histidine	4-CH (ring)	7.10(s)	117.8
		2-CH (ring)	7.89(s)	136.4
		5-C (ring)		131.4
36	tryptophan	5-CH (ring)	7.20(t)	120.0
		6-CH (ring)	7.28(t)	122.6
		2-CH (ring)	7.33(s)	125.7
		7-CH (ring)	7.54(d)	112.5
		4-CH (ring)	7.73(d)	119.1
		3-C (ring)		108.1
		8-C (ring)		137.0
37	phenylalanine	2,6-CH (ring)	7.34(m)	129.9
		4-CH (ring)	7.39(m)	128.4
		3,5-CH (ring)	7.43(m)	129.6
		1-C (ring)		135.8
38	N-methylnicotinate (trigonelline, NMNA)	CH_3	4.44(s)	48.8
		5-CH	8.09(#)	#
		4-CH	8.84(#)	#
		6-CH	8.85(#)	#
		2-CH	9.13(s)	#
39	nicotinamide mononucleotide (nicotinamide ribotide, NMN)	5-CH	8.33(t)	#
		4-CH	9.00(d)	#
		6-CH	9.35(d)	#
		2-CH	9.59(s)	#
40	formate	CH	8.46(s)	#
41	β-D-fructopyranose	1-CH	#	#
		2-C		98.3
		3-CH	#	#
		4-CH	#	#
		5-CH	#	#
		half 6-CH_2	3.72(#)	#
		half 6-CH_2	4.03(dd)	64.0
42	β-D-fructofuranose	1-CH	#	#
		2-C		104.7
		3-CH	4.12(d)	76.2
		4-CH	4.12(d)	75.1
		5-CH	3.85(#)	#
		half 6-CH_2	3.69(#)	#
		half 6-CH_2	3.82(#)	#

[a] multiplicity for ¹H resonances: s, singlet; d, doublet; t, triplet; q, quartet; qu, quintet; m, multiplet; dd, doublet of doublets; #, multiplicity can't be determined. [b] #: signals were not determined.

Table 2. OPLS-DA loadings correlation coefficients.

Metabolite (chemical shift)	Keys	Coefficients (r)		
		M vs. WT	ECE vs. WT	ECE vs. M
Amino acids & derivatives				
γ-aminobutyrate (GABA, δ 2.30)	9	0.850	0.888	0.689
glutamate (δ 2.36)	10	-0.743	-0.807	
glutamine (δ 2.46)	11		0.749	0.789
isoleucine (δ 1.02)	1	-0.771	-0.753	
leucine (δ 0.97)	2	-0.767		
valine (δ 1.05)	3	-0.756	-0.653	
ethanolamine (δ 3.15)	19	-0.805	-0.874	-0.775
choline (δ 3.21)	20	0.611		
phosphocholine (δ 3.23)	21		-0.882	-0.787
threonine (δ 1.34)	5	-0.768	-0.786	
aspartate (δ 2.69)	17	-0.835	-0.760	
asparagine (δ 2.87)	18		-0.704	-0.674
alanine (δ 1.49)	7		0.829	0.789
arginine (δ 1.66)	8		-0.779	-0.747
histidine (δ 7.10)	35	-0.803	-0.890	-0.771
phenylalanine (δ 7.43)	37	-0.837	-0.864	-0.732
tryptophan (δ 7.28)	36	-0.742	-0.872	-0.757
tyrosine (δ 6.91)	34	-0.940	-0.927	-0.821
Carbohydrate metabolites & TCA cycle intermediates				
sucrose (δ 5.42)	26		-0.878	-0.916
glucose (δ 5.25)	23	0.847	0.848	0.768
fructose (δ 4.12)	42	0.767	0.803	0.743
succinate (δ 2.41)	13	-0.795		0.712
fumarate (δ 6.53)	33	-0.843		0.823
malate (δ 4.30)	12	-0.863		0.692
citrate (δ 2.54)	15	-0.821		
Nucleotide metabolites				
adenosine (δ 8.35)	31		0.712	
adenosine monophosphate (AMP) (δ 8.61)	32		-0.714	
uridine (δ 5.91)	27	0.714	-0.632	-0.662
Other metabolites				
lipid (δ 1.30)	4	0.860	0.838	0.651
N-methylnicotinate (trigonelline, NMNA, δ 9.13)	38	0.842	0.766	
formate (δ 8.46)	40		0.848	0.813
mono-methyl phosphate (δ 3.49)	25	0.804	-0.712	-0.903

WT, the wild type; M, the *oscyp96b4* semi-dwarf mutant; ECE, the *OsCYP96B4* ectopic expression.

Table 3. Primers for quantitative real-time PCR analysis on selected genes.

Accession	Gene Name	Primers (5′-3′)		Product length (bp)
		Forward Sequence	Reverse Sequence	
LOC_Os02g14110	aspartate aminotransferase, AAT	AGAGGCGTTTCTTGCCAAATT	CCATCAGTCCTGCGAAATCA	150
LOC_Os03g28330	sucrose synthase 1, SUS1	TTGTGCAGCCCGCTTTCTAC	CACCCTGGTATGGGTCAATGT	150
LOC_Os05g48200	glutamate synthase 2, GOGAT2	GTCACGAAGGACGCAGGTTT	CCAGCCGAGGACGGTATG	150
LOC_Os01g52230	phosphoethanolamine/phosphocholine phosphatase, PHOSPHO1	CGGCCTCACGGGTTACTTCT	CTGGCCCTTGCACATGTTGG	154
LOC_Os02g50240	glutamine synthetase, GS	GGAACGATGGTGGCTACGA	CTCCCCAGCTGAAGGTGTTG	150
LOC_Os04g42520	adenine phosphoribosyltransferase, APRT	ACCGTTGCGCAAACCTAAGA	CCACCGGTTGCAACTAAATCA	150
LOC_Os04g46560	malate dehydrogenase, MDH	CTTCCAGCGTCTGGCAAAAT	CGGCTGGTCGGCTCCTA	150
LOC_Os08g09200	Aconitase, ACO	ATGGAGAAGTTGGACCCAAGA C	CAGCCCAGTCACGAGAACTG	150
LOC_Os09g12290	aspartate kinase, AK	GGCTCGAGCTTTCCGATATTC	GCACTTCTCCTGCAGCTTCAG	150
LOC_Os09g20440	succinate dehydrogenase, SDH	CCGCGTCGAAGTTGCTTAC	ACCAACCCAACTGCATGGA	150
LOC_Os10g25140	alanine transaminase, ALT	TCGTGGCGCAACTCTTGTAC	CCAGTAGGGTTTCCCGGATTT	150
LOC_Os01g09460	hexokinase-8, HXK8	AGCATGTCGTCAACTCTGAATC C	TCTTGCCGTTTGGCATGTC	150
LOC_Os02g16630	phosphoribosylanthranilate isomerase, PRAI	AATGCCAATGACGACGGAAA	CCCATTCTTGCTTCGAACAGA	150
LOC_Os02g19970	tyrosine aminotransferase, TAT	TCCAAGCGATCGAAGTCATG	CCCAGCCTCTCTCTGGAATG	150
LOC_Os03g09910	LL-diaminopimelate aminotransferase, LL-DAP-AT	TGCTGCGCAATCCAAACAT	CGATGCTTGGTATGGGTTCA	150
LOC_Os03g50480	phosphoglucomutase, PGM	GTGATGCTGATCGCAACATGA	CAGCTGATGTGGGCATGCT	150
LOC_Os03g50880	anthranilate synthase beta subunit 2, ASB2	GCCCTGGCACACCTCAAG	GCACAACTCCATAAGGAGAAC GA	150

LOC_Os04g37500	glutamate decarboxylase 4, GAD4	CCACAAGTACGGGCTCGTCTAC	ATCTGACTGGACCCTTTGGAGAA	150
LOC_Os04g40950	glyceraldehyde 3-phosphate dehydrogenase, GAPDH	TCACCTGAAGGGTGGTGCTAA	AGTGGAGCAAGGCAGTTGGT	150
LOC_Os04g58390	allantoinase, ALN	CAGGCGTCTTAGGGCTCAAG	GAACATCGGGTATGCGTTCTG	150
LOC_Os05g33380	fructose-bisphosphate aldolase, FBA	CCCTGTACCAGAAGACCAAGGA	GCGCATCTCTTGCCAAGGT	150
LOC_Os05g40420	2,3-bisphosphoglycerate-independent phosphoglycerate mutase, iPGM	CACTTGGTGCTGGTCGGATT	CGTCGCTCAACAACCCAATA	150
LOC_Os08g23150	indole-3-glycerol phosphate synthase, IGPS	AGCCTTCAAGCGCAATGGTA	TCGGTCAAGATGCTCAAGCA	150
LOC_Os08g32870	betaine aldehyde dehydrogenase 2, BADH2	GAATCCTTGGACAAAAGGCAAA	GGCAGGAGCTACCTTCCATGT	150
LOC_Os03g50885	actin-1, ACT1	CCAAGGCCAATCGTGAGAAGA	AATCAGTGAGATCACGCCCAG	228

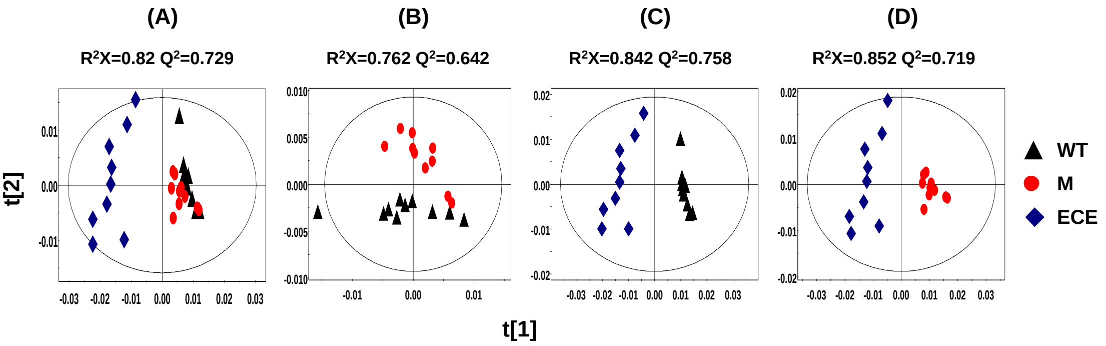

Figure S1. PCA scores plots derived from ^{1}H NMR spectra of rice plant extracts from different groups. (**A**) the wild type (WT, ▲), the *oscyp96b4* semi-dwarf mutant (M, ●), and the *OsCYP96B4* ectopic expression (ECE, ◆) rice lines, the first and second principal component contains 64.3% and 17.7% of the variance respectively. (**B**) the wild type (WT, ▲) and the *oscyp96b4* semi-dwarf mutant (M, ●) rice lines, the first and second principal component contains 54.2% and 22.0% of the variance respectively. (**C**) the wild type (WT, ▲) and the *OsCYP96B4* ectopic expression (ECE, ◆) rice lines, the first and second principal component contains 65.2% and 19.0% of the variance respectively. (**D**) the *oscyp96b4* semi-dwarf mutant (M, ●) and the *OsCYP96B4* ectopic expression (ECE, ◆) rice lines, the first and second principal component contains 64.7% and 20.5% of the variance respectively.

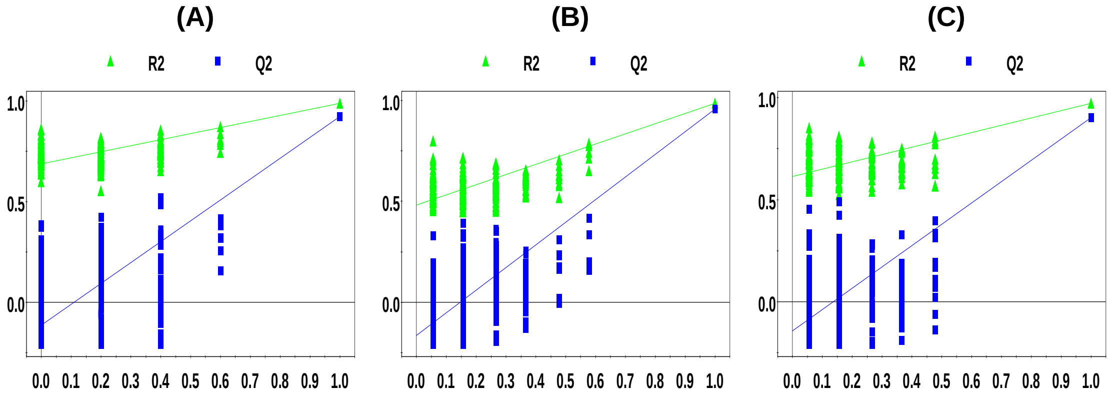

Figure S2. Permutation test results (with 200 permutations) for PLS-DA models (with 2 components) derived from ^{1}H NMR spectra of rice plant extracts from different groups. (**A**) the wild type (WT) and the *oscyp96b4* semi-dwarf mutant (M) rice lines, intercepts: R^2 = (0.0, 0.688), Q^2 = (0.0, -0.114); (**B**) the wild type (WT) and the *OsCYP96B4* ectopic expression (ECE) rice lines, intercepts: R^2 = (0.0, 0.481), Q^2 = (0.0, -0.164); (**C**) the *oscyp96b4* semi-dwarf mutant (M) and the *OsCYP96B4* ectopic expression (ECE) rice lines, intercepts: R^2 = (0.0, 0.613), Q^2 = (0.0, -0.144).

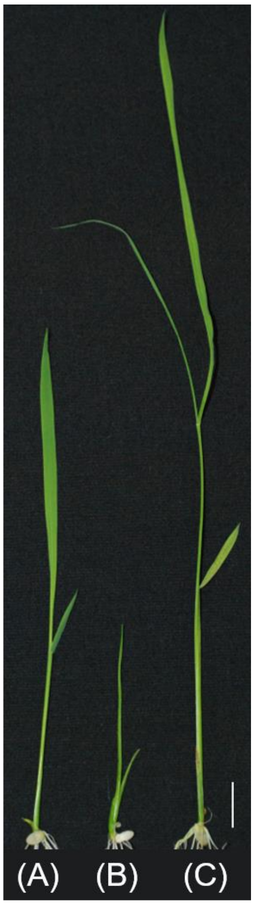

Figure S3. Representative phenotypes of the 2-week-old (A) *oscyp96b4* semi-dwarf mutant (M), (B) *OsCYP96B4* ectopic expression (ECE) and (C) wild-type (WT) rice plants. Scale bar = 1 cm.
